# The association between EPCR gene p.Ser219Gly polymorphism and venous thromboembolism risk: a case–control study, meta-analysis, and a reproducibility study

**DOI:** 10.3389/fcvm.2023.1270093

**Published:** 2023-11-22

**Authors:** Dóra Pituk, Tünde Miklós, Ágota Schlammadinger, Katalin Rázsó, Zsuzsanna Bereczky

**Affiliations:** ^1^ Division of Clinical Laboratory Sciences, Department of Laboratory Medicine, Faculty of Medicine, Kálmán Laki Doctoral School, University of Debrecen, Debrecen, Hungary; ^2^ Division of Clinical Laboratory Science, Department of Laboratory Medicine, Faculty of Medicine, University of Debrecen, Debrecen, Hungary; ^3^ Division of Hematology, Department of Internal Medicine, Faculty of Medicine, University of Debrecen, Debrecen, Hungary

**Keywords:** endothelial protein C receptor, venous thrombosis, *PROCR* gene, Ser219Gly, rs867186, polymorphism, single nucleotide, meta-analysis

## Abstract

**Background:**

The rs867186 single-nucleotide polymorphism in the *PROCR* gene (g.6936A > G, c.4600A > G) results in a serine-to-glycine substitution at codon 219 of endothelial protein C receptor (EPCR). We performed a case–control study followed by an updated meta-analysis of the association between this polymorphism and the risk of venous thromboembolism (VTE).

**Objective and methods:**

We enrolled 263 VTE patients and 320 unrelated healthy controls for the case–control study. The total number of cases and controls for the meta-analysis were 5,768 and 30,017, respectively. A new online MetaGenyo Statistical Analysis System software was used to perform the current meta-analysis. Furthermore, a reproducibility study was conducted to validate our results.

**Results:**

Among well-defined thrombosis risk factors, Factor V Leiden was more frequent in the VTE group (*p* < 0.001), while there was no difference in mutation frequency of prothrombin 20210G>A polymorphism between the two groups. There was no difference in the mutation frequency of Factor V Leiden and prothrombin 20210G>A between cases with and without provoking factors and cases with and without VTE recurrence. The rs867186 “G” carriership did not influence the risk of VTE [odds ratio (OR) 1.339; 95% confidence interval (CI): 0.904–1.984] in our study. No significant differences could be demonstrated among the rs867186 genotype frequencies between VTE cases with and without provoking factors (*p* = 0.430). *PROCR* rs867186 was associated with an OR of 1.72 (95% CI: 0.95–3.13, *p* = 0.075) in terms of VTE recurrence. In the meta-analysis, a significant association was found between EPCR Ser219Gly polymorphism and VTE under the dominant model (OR = 1.27, 95% CI: 1.11–1.46, *p* = 0.0006), the recessive model (OR = 1.60, 95% CI: 1.26–2.04, *p* = 0.0001), the GG vs. AA contrast model (OR = 1.64, 95% CI: 1.28–2.09, *p* = 0.0001), and the GA vs. AA contrast model (OR = 1.24, 95% CI: 1.08–1.43, *p* = 0.002).

**Conclusion:**

The rs867186 was not associated with the first VTE risk in our case–control study; however, a tendency to VTE recurrence was observed. Based on the results of our reproducibility study, MetaGenyo is acceptable for meta-analysis in case of genetic epidemiology studies. Although the risk conferred by the rs867186 is mild in all meta-analyses, including ours, identifying patients carrying the minor allele might have an impact on personalized VTE risk assessment, risk-score calculation, and patient management.

## 1. Introduction

Two possible manifestations of venous thromboembolism (VTE) are deep venous thrombosis (DVT) and pulmonary embolism (PE) (1). VTE is a common cardiovascular disease associated with high mortality; accurately, this condition is the third most common cause of death worldwide (2). Based on the American Heart Association (AHA) Heart Disease and Stroke Statistics—2023 report, in the United States, there were approximately 1,036,000 total VTE cases in 2019 (the US population was 328 million in 2019) (3). The incidence rate for VTE varies in European countries, mainly 1.4–3.2 per 1,000 person-years (4). Intrinsic and environmental risk factors are recognized in VTE patients, especially since there are several well-established pathogenic mutations in antithrombin (AT), Protein C (PC), and Protein S (PS) genes (*SERPINC1, PROC*, and *PROS1*, respectively) that are suggested to influence the risk of thrombosis and the levels of these natural anticoagulants (5–9). While AT, PC, and PS deficiencies are rare but strong risk factors of VTE, they are included in the routine thrombophilia investigation protocol; the investigation of the endothelial protein C receptor (EPCR)—either its plasma level or the genetic variations in the *PROCR* gene—is currently not part of the risk assessment. VTE is a typical example of a common complex disease in which several genetic determinants may play a role, and gene–gene and gene–environment interactions have been explored in its background. Therefore, individualized risk stratification is a rational approach to establish the patient's personal thrombosis risk and the risk of recurrence. In the case of such a complex disease, the amount of risk conferred by the individual genes may not be strong one by one; however, these variations may play an enhancing effect on the development of VTE. Moreover, studies on the association of these common gene variations (having minor allele frequency, MAF, of at least 0.1) with VTE may help in the estimation of the risk at the population level.

Protein C (PC) is a natural, endogenous anticoagulant protein with anti-inflammatory properties, which interacts with the EPCR (10). The EPCR enhances the activation of protein C on the endothelial cell surface. In contrast to this transmembrane EPCR, the soluble form (sEPCR) lacks the transmembrane domain and has an opposite effect to that of the transmembrane form. The sEPCR inhibits the activation of PC and also the anticoagulant activity of the activated PC (APC) (11). There are single-nucleotide polymorphisms (SNPs), which affect the levels of circulating protein C. The rs867186 diallelic single-nucleotide polymorphism in the endothelial protein C receptor (*PROCR*) gene (20q11) causes a p.Ser219Gly substitution (NC_000020.11:g.35176751 A>G, c.655 A>G, p.Ser219Gly, MAF 0.10), which increases the solubility relative to membrane-bound EPCR levels and explains 75% of the variability of plasma-soluble EPCR levels and also has an effect on PC levels (12).

Recently, the results of genome-wide association (GWA) studies are also available (13). The MARTHA project described a significant association of *PROCR* rs867186, rs6060278 (NC_000020.11:g.35165459 T > C, MAF 0.23), and rs8119351 (NC_000020.11:g.35166602 G > A, MAF 0.09) with PC levels in VTE patients explaining 20% of the variance (14). The rs6060278 was in strong negative Linkage disequilibrium (LD) (*r*^2^ = 0.03, D′ = −1) with the p.Ser219Gly according to the study by Germain et al. (15). The adjusted odds ratio (OR) for venous thrombosis (VT) was 1.33 [95% confidence interval (CI): 1.11–1.60] in the presence of the “G” allele of rs867186 in their study. They also found that *PROCR* rs6088735 (NC_000020.11:g.35157873 C>T, MAF 0.22) elevated the risk of VTE. *PROCR* rs8119351 G>A leads to elevated PC levels, but its mechanism is unknown (16). While the rs867186 “A” allele could indicate haplotypes H1, H2, or H4, the rs867186 “G” allele exclusively associates with the H3 haplotype of EPCR. The p.Ser219Gly leads to an increased level of soluble EPCR, reflecting an increased shedding of EPCR from the endothelial membrane (17). The mechanism underlying the association between the EPCR p.Ser219Gly variant and higher soluble EPCR and variation in plasma protein C level is unclear (13). Several previously published studies (18–27) investigated the connection between EPCR gene p.Ser219Gly gene polymorphism and VTE risk with heterogeneous results, and furthermore, two meta-analyses (13, 28) were conducted.

Recently, evidence for an association between the EPCR gene p.Ser219Gly gene polymorphism and venous thrombosis is still conflicting. Previously published studies showed that rs6088735 and rs867186 could act additively on the risk of VTE (15, 29).

Therefore, this population-based case–control study and an updated, comprehensive meta-analysis aimed to investigate the potential relationship between EPCR gene p.Ser219Gly gene polymorphism and VTE risk.

## 2. Materials and methods

### 2.1. Recruitment of the study population

Our study population consisted of non-related individuals with VTE in their case histories (*n* = 263) and of apparently healthy unrelated adults (control subjects, *n* = 320).

The Division of Clinical Laboratory Science, Department of Laboratory Medicine, Faculty of Medicine, University of Debrecen, recruited the VT cases consecutively during a 1-year period between February 2014 and January 2015. Diagnosis of VT was established by standard diagnostic modalities, such as color Doppler ultrasound or phlebography at the Department of Internal Medicine. Thrombosis was described according to the guidance of the International Society of Thrombosis and Haemostasis (30). Briefly, acquired risk factors that occurred within 1 month before the diagnosis of VTE as trauma, surgery, hospitalization due to acute illness, central venous catheters, immobilization, pregnancy, oral anticoncipient use, hormonal treatment, prolonged travel, L-asparaginase treatment, the presence of positive family history, and the presence of acquired risk factors [i.e., chronic situations such as malignancy, paroxysmal nocturnal hemoglobinuria, autoimmune diseases, antiphospholipid syndrome, body mass index (BMI), varicose veins, nephrotic syndrome, heart failure, and long-term immobilization] were registered.

Healthy volunteers recruited by our study group in the same period of time were free from any known hemostasis diseases (e.g., hereditary hemorrhagic diathesis and acquired hemorrhagic disease) or drugs influencing hemostasis (vitamin K antagonist, heparin, novel oral anticoagulants, etc.); there was no venous or arterial thrombosis in their case histories (deep vein thrombosis and pulmonary embolism, myocardial infarction, ischemic stroke, and peripheral arterial disease).

Individuals with antithrombin, protein C, or protein S deficiencies with causative mutations in the background were excluded from the whole study population. Individuals with chronic illnesses (autoimmune, malignancies, chronic inflammation, renal, or hepatic diseases) were also excluded. Hypertension, diabetes mellitus, and their therapy were allowed in the study population. We also let in women taking oral anticontraceptive pills (OAC).

The work was carried out according to the principles laid down in the Declaration of Helsinki and amended in 2008 by the World Medical Assembly in Seoul, Korea. Ethical approval for the study was obtained from the National Ethical Council (3166/2012/HER), and informed consent was obtained from all participants.

### 2.2. Blood sampling and SNP genotyping

Blood samples were collected from the antecubital vein into vacutainer tubes (Beckton Dickinson, Franklin Lakes, NJ, USA) with anticoagulant (1/10 volume of 0.109 M citrate). DNA was isolated from the buffy coat of citrated blood samples by QIAamp DNA Blood Mini Kit (Qiagen, Hilden, Germany). Factor V Leiden mutation (FVL) and prothrombin (FII) 20210 G>A mutation were analyzed by standard molecular genetic methods (31, 32).

Twelve SNPs, located in the *PROC, PROCR, PROS1*, and *SERPINC1* genes were analyzed using the ABI PRISM SNaPshot Multiplex Kit (Applied Biosystems, Foster City, CA, USA), among which only the effect of EPCR p.Ser219Gly (rs867186) was investigated in this study. The effects of two other *PROCR* polymorphisms, namely, rs8119351 and rs6088735, were also taken into consideration. Using this procedure, we identified the SNPs by utilizing fluorescently labeled dideoxynucleotides (ddNTPs) during the elongation of template-specific primers in thermocycling. PCR primers for multiplex reactions (provided upon request) and the single base extension primers (SNP-specific primers) were designed to have an annealing temperature of 58°C using Primer3Plus free online software. Primers were synthesized by Integrated DNA Technologies (Munich, Germany). After PCR amplification and purification, multiplex minisequencing was performed in a 10 µl reaction volume using 3 µl purified PCR products, 2 µl SNP-specific primers, and 5 µl of SNaPshot ready reaction mix. Sequence cycling consisted of 40 cycles of denaturation at 96°C for 10 s, primer annealing at 50°C for 5 s, and primer extension at 60°C for 30 s. The amplified products were treated with 0.7 µl SAP (shrimp alkaline phosphatase, Sigma-Aldrich, Steinheim, Germany) to prevent further binding of ddNTPs. The mixture was incubated for 60 min at 37°C followed by 15 min incubation at 75°C. The SAP-treated products (0.5 µl) were mixed with 9 µl HiDi formamide (Life Technologies, Carlsbad, CA, USA) and 0.5 µl of GeneScan 120-LIZ size standard (Applied Biosystems, Foster City, CA, USA), denatured at 95°C for 5 min, and kept on ice for at least 1 min. The fluorescently labeled fragments were separated in POP7 polymer on an ABI 3130 Genetic analyzer (Applied Biosystems). Data analyses were performed using GeneMapper Software v4.1. The detailed protocol is available upon request.

Thrombophilia investigation including plasma assays for antithrombin, protein C, and protein S deficiencies were performed using Siemens reagents (Innovance AT, Berichrom PC, Innovance free PS) on a BCS-XP automated coagulometer (Siemens, Marburg, Germany). Plasma was separated by centrifugation at 1,500 g for 20 min, and 500 μl aliquots were stored at −70°C until determination.

### 2.3. Statistical analysis for case–control study

The differences in the distribution of demographic characteristics and genotypic frequencies of *PROCR* rs867186 polymorphism between the cases and controls were examined by using the *χ*^2^ test. To assess the associations between *PROCR* rs867186 polymorphism and VTE risk, unconditional logistic regression for crude OR with 95% CI and adjusted OR with 95% CI were calculated. Continuous variables were expressed as median, minimum–maximum values, and interquartile ranges (IQR). IQR is the region between the 25th and 75th percentile. The statistical analysis for the case–control study was performed using Statistical Package for Social Sciences (SPSS version 23.0, Chicago, IL, USA) software and Stata 17 (Statistical Software: Release 17; Stata Corp LLC, College Station, TX, USA). A *p*-value of 0.05 or less was considered to indicate statistical significance.

### 2.4. Meta-analysis

Meta-analysis was executed on high methodology quality based on the recommended list of the Preferred Reporting Items for Systematic Review and Meta-analyses (PRISMA) (33).

#### 2.4.1. Meta-analysis—literature search strategy

A primary search was conducted to find all relevant studies through MEDLINE using the following terms of combination, including “6936A/G,” “rs867186,” “4600AG,” “thromboembolism,” “thrombosis,” “EPCR,” and “*PROCR*.” Only those studies that investigated the association between EPCR gene polymorphism and venous thromboembolism risk were included in our meta-analysis. This current search was done through May 2023, and our search strategy had no language restrictions.

#### 2.4.2. Eligibility criteria—inclusion and exclusion criteria

All the selected studies met the following criteria: (1) available studies investigate the association between EPCR gene polymorphism and venous thromboembolism risk; (2) case–control or cohort studies; (3) the studies have proper allele and/or genotype frequency information for cases and controls. The exclusion criteria were the following: (1) no properly provided information about the predictor and the outcome variable; (2) reviews, abstracts, or conference materials; (3) duplicated data.

#### 2.4.3. Data extraction

Two authors were responsible for the literature screening and the data extraction procedure from the included studies. The following data were extracted from all included studies: first author, publication year, sample size and the number of cases and controls, ethnicities, additional pieces of information on the study population (age, gender, matching strategy), genotype, and allele distribution of cases and controls.

#### 2.4.4. Data synthesis and statistical analysis for meta-analysis and reproducibility study

Based on the different genetic models, the ORs and their corresponding 95% CIs were used to determine the association between *PROCR* rs867186 polymorphism and venous thromboembolism risk. We analyzed the following genetic models: AG vs. AA, GG vs. AA, GG + AG vs. AA (dominant model), GG vs. AG + AA (recessive model), and G vs. A (allele contrast). The MAF was considered for the G allele. To analyze between-study heterogeneity, the Cochran *Q*-statistic and *I*^2^-statistic were used. *I*^2^ value was used to describe the percentage of variation across studies due to heterogeneity. *I*^2^ values of 25%, 50%, and 75% show low, medium, and high between-study heterogeneity, respectively (34). Cochran's *Q* Test *p* > 0.1 was considered not statistically significant heterogeneity (35, 36). The inverse variance-weighted effects meta-analysis was used if no statistically significant heterogeneity was observed; otherwise, the random-effect model (DerSimonian–Laird method) was applied (37, 38). The sensitivity analysis was performed to determine the individual effect of each selected study on the pooled analysis. Egger's test assessed the publication bias, and it was visualized by a funnel plot (39). The significance level was set at *p* < 0.05. The MetaGenyo web tool (40) was used to perform the statistical analysis for meta-analysis. In order to evaluate the reliability of MetaGenyo, we also performed a reproducibility study in which we compared the results obtained by the MetaGenyo web tool with those generated by the STATA statistical software performed by Dennis et al. previously (13). In the reproducibility study, we demonstrated the deviation in percentage between the results obtained by the MetaGenyo and STATA platforms. The compared parameters were as follows: the deviation from Hardy–Weinberg equilibrium (HWE), the test of association (OR and 95% CI), and between-study heterogeneity (*I*^2^ and Cohran *Q p*-value).

## 3. Results

### 3.1. Case–control study

After informed consent, *n* = 263 VTE cases and *n* = 320 unrelated healthy control patients were included in the study (Table 1A). Both VTE cases and controls fell within the age range of 18–85 years, and median age of VTE cases was significantly higher as compared to controls. There was no difference in gender distribution between the two groups. BMI and the frequency of positive family history of thrombosis were significantly higher in the VTE group. The frequency of smokers was significantly lower among cases. The numbers of individuals among females taking oral contraceptives were *n* = 18 in the cases group and *n* = 17 in the control group, in which the difference was not statistically significant. Within the VTE group, 23% had any provoking factors related to thrombosis. Among them, 30% were OAC users, 23.3% had trauma and/or plaster cast in their case histories, 16.6% had surgery, 11.7% had thrombosis in the postpartum period, 5% had long travel before thrombosis, immobilization was registered in 5% of cases, 3.3% had pregnancy-associated thrombosis, 1.7% had hormonal replacement, 1.7% had varicose veins, and 1.6% had thrombosis related to intravenous cannula. Cases with provoking factors in their case histories were significantly younger, and the ratio of females was significantly higher compared to cases without provoking factors (Table 1B). There were no differences between the two groups in terms of smoking, BMI, frequency of positive family history, and recurrent VT and PE. Recurrent VT was found in 28% and the frequency of PE was 27% in the VTE group. Frequencies of diabetes mellitus and hypertension were significantly higher in the cases group; however, there were no differences in these two variables between cases with and without provoking factors of VT. FV Leiden mutation was more frequent in the VTE group (*p* < 0.001), while there was no difference in mutation frequency of prothrombin 20210G>A polymorphism between the two groups. There was no difference in the mutation frequency of FV Leiden and prothrombin 20210G > A polymorphism between cases with and without provoking factors.

**Table 1A:** Demographic and clinical characteristics of the case–control study population.

Characteristics	Cases (*n* = 263)	Controls (*n* = 320)	*p*-value
Age, years (median; min–max); IQR	45 (18–84); 25	39 (18–85); 21	<0.001
Male/female (*n*)	135/128	141/179	0.067
Body mass index (kg/m^2^) (median, min–max); IQR	29 (18–53); 7	25 (16–41); 7	<0.001
Smoking, %	13	27	<0.001
Hypertension, %	28	19	0.013
Diabetes mellitus, %	9	1	<0.001
Positive family history, %	29	17	<0.001
OAC (oral anticoncipient), %	14	10	0.275
Provoking factors % within VT patients	23	—	—
Recurrent VT % within VT patients	28	—	—
PE % within VT patients	27	—	—
MI or stroke % within VT patients	4	—	—
FV Leiden (rs6025) WT; HeZ; HoZ%	65; 30; 5	92; 8; 0	<0.001
PT 20210G>A (rs1799963) WT; HeZ; HoZ%	94; 6; 0	97; 3; 0	0.107
PROCR G>A (rs8119351) WT; HeZ; HoZ%	80; 19; 1	82; 17; 1	0.572
PROCR C>T (rs6088735) WT; HeZ; HoZ%	56; 38; 6	59; 37; 4	0.536
PROCR A>G (rs867186) WT; HeZ; HoZ%	75; 24; 1	81; 18; 1	0.314

**Table 1B:** Demographic and clinical characteristics of the cases according to the presence or absence of provoking factors.

Characteristics	Cases with provoking factors (*n* = 61)	Cases without provoking factors (*n* = 202)	*p*-value
Age, years (median; min–max); IQR	41 (19–73); 21	47 (18–84); 26	0.019
Male/female (*n*)	22/39	113/89	0.006
Body mass index (kg/m^2^) (median, min–max); IQR	29 (19–45);7	29 (18–53); 7	0.244
Smoking, %	15	12	0.628
Hypertension, %	24	29	0.482
Diabetes mellitus, %	8	9	0.774
Positive family history, %	34	27	0.324
Recurrent VT %	20	31	0.174
PE %	25	27	0.683
MI or stroke %	2	4	0.314
FV Leiden (rs6025) WT; HeZ; HoZ%	60; 30; 10	66; 30; 4	0.130
PT 20210G>A (rs1799963) WT; HeZ; HoZ%	98; 2; 0	93; 7; 0	0.098
PROCR G>A (rs8119351) WT; HeZ; HoZ%	82; 18; 0	79; 20; 1	0.593
PROCR C>T (rs6088735) WT; HeZ; HoZ%	52; 40; 8	57; 37; 6	0.713
PROCR A>G (rs867186) WT; HeZ; HoZ%	80; 20; 0	74; 25; 1	0.430

MI, Myocardial infarction; WT, wild-type; HeZ, heterozygous form; HoZ, homozygous form.

The p.Ser219Gly polymorphism and the rs6088735 and rs8119351 in the study population met the criteria of Hardy–Weinberg equilibrium (*p* = 0.7687, *p* = 0.5521, *p* = 0.6701, respectively). The three SNPs showed a strong LD in our study population. Upon investigating the combination of the three SNPs, the most frequent haplotype was “ACG” (frequency 0.659), followed by “ATG” (frequency 0.232), while haplotypes “GCA” (frequency 0.094) and “GCG” (frequency 0.014) were rare variants.

In the whole study population, without any adjustments or subgroup analysis, no significant differences could be demonstrated among the rs867186 genotype frequencies between VTE cases and controls and between cases with and without provoking factors (Tables 1, 2) Subgroup analysis according to either gender or smoking did not show any significant difference in rs867186 genotype frequencies. In the subgroups of individuals with BMI less than 30 kg/m^2^ or age younger than 60 years (below the 75th percentile of VTE cases), the number of wild-type and mutation carriers of rs867186 did not differ significantly. If only rs6025 (FV Leiden) or rs1799963 (FII2021G>A) wild-type or carrier individuals were taken into consideration, no significant differences in genotype frequencies of the rs867186 were demonstrated. We analyzed our study group in terms of VTE recurrence and *PROCR* rs867186 and we found an OR of 1.72 (95% CI: 0.95–3.13; *p* = 0.075), which remained similar even in the adjusted model. FV Leiden and FII 20210A did not influence VTE recurrence risk in our analysis.

**Table 2:** Comparison of the distribution of rs867186 in cases and controls in the whole study population and in different subgroups.

Category/subgroup	Genetic comparison	Cases	Control	OR	95% CI	*p*-value
Overall	AA vs. AG + GG^a^	198/65	257/63	1.339	0.904–1.984	0.145
	AG + AA vs. GG^b^	260/3	316/4	0.912	0.202–4.109	0.904
Female	AA vs. AG + GG^a^	98/30	147/32	1.406	0.803–2.461	0.233
	AG + AA vs. GG^b^	127/1	177/2	0.697	0.063–7.768	0.769
Male	AA vs. AG + GG^a^	100/35	110/31	1.242	0.714–2.161	0.443
	AG + AA vs. GG^b^	133/2	139/2	1.045	0.145–7.527	0.965
Smokers	AA vs. AG + GG^a^	27/7	67/17	1.022	0.381–2.742	0.966
	AG + AA vs. GG	33/1	82/2	1.242	0.109–14.172	0.861
BMI (less than 30 kg/m^2^)	AA vs. AG + GG^a^	115/34	224/51	1.299	0.797–2.117	0.295
	AG + AA vs. GG^b^	147/2	271/4	0.922	0.167–5.092	0.926
Age (younger than 60 years)	AA vs. AG + GG^a^	149/52	237/59	1.402	0.916–2.145	0.120
	AG + AA vs. GG^b^	200/1	292/4	0.365	0.040–3.290	0.369
FV (rs6025) wild type	AA vs. AG + GG^a^	133/38	232/58	1.143	0.721–1.813	0.570
	AG + AA vs. GG^b^	169/2	286/4	0.846	0.153–4.669	0.848
FV (rs6025) mutant	AA vs. AG + GG^a^	65/27	23/5	1.911	0.658–5.549	0.234
	AG + AA vs. GG^b^	91/1	28/0	NA	NA	NA
FII (rs1799963) wild type	AA vs. AG + GG^a^	185/62	247/61	1.357	0.908–2.028	0.136
	AG + AA vs. GG^b^	244/3	305/3	1.250	0.250–6.248	0.786
FII (rs1799963) mutant	AA vs. AG + GG^a^	13/3	9/1	2.077	0.185–23.298	0.553
	AG + AA vs. GG^b^	16/0	10/0	NA	NA	NA

NA, not applicable.

^a^ Dominant model: AA vs. AG + GG.

^b^ Recessive model: AG + AA vs. GG.

Since the vast majority of our patients were on vitamin K antagonist therapy at the time of recruitment and no therapy modification was allowed during the study; protein C (PC) anticoagulant activity could not be interpreted in them. However, in healthy individuals, who were not on anticoagulant therapy, we demonstrated that the presence of rs867186 increased the PC activity significantly having extremely high plasma PC activity in homozygote mutants. In wild-type individuals, median PC activity was 112% (IQR24); in heterozygotes, median PC activity was 128.5% (IQR32); and in homozygotes (*n* = 3), median PC activity was 160%.

### 3.2. Meta-analysis

**Figure 1:**
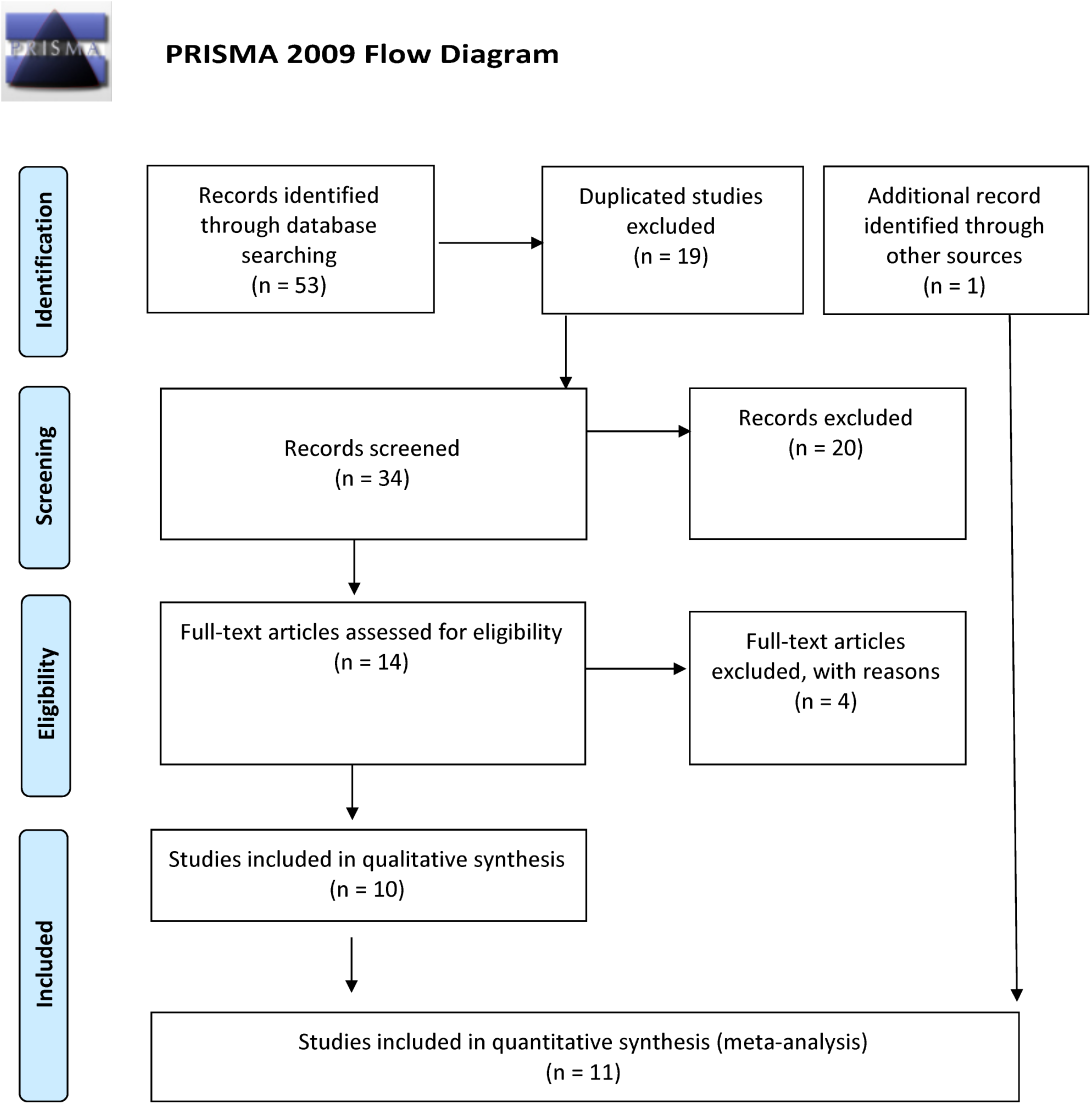
PRISMA 2009 flow diagram showing the selection process for the PROCR rs867186 polymorphism and venous thromboembolism.

**Figure 2:**
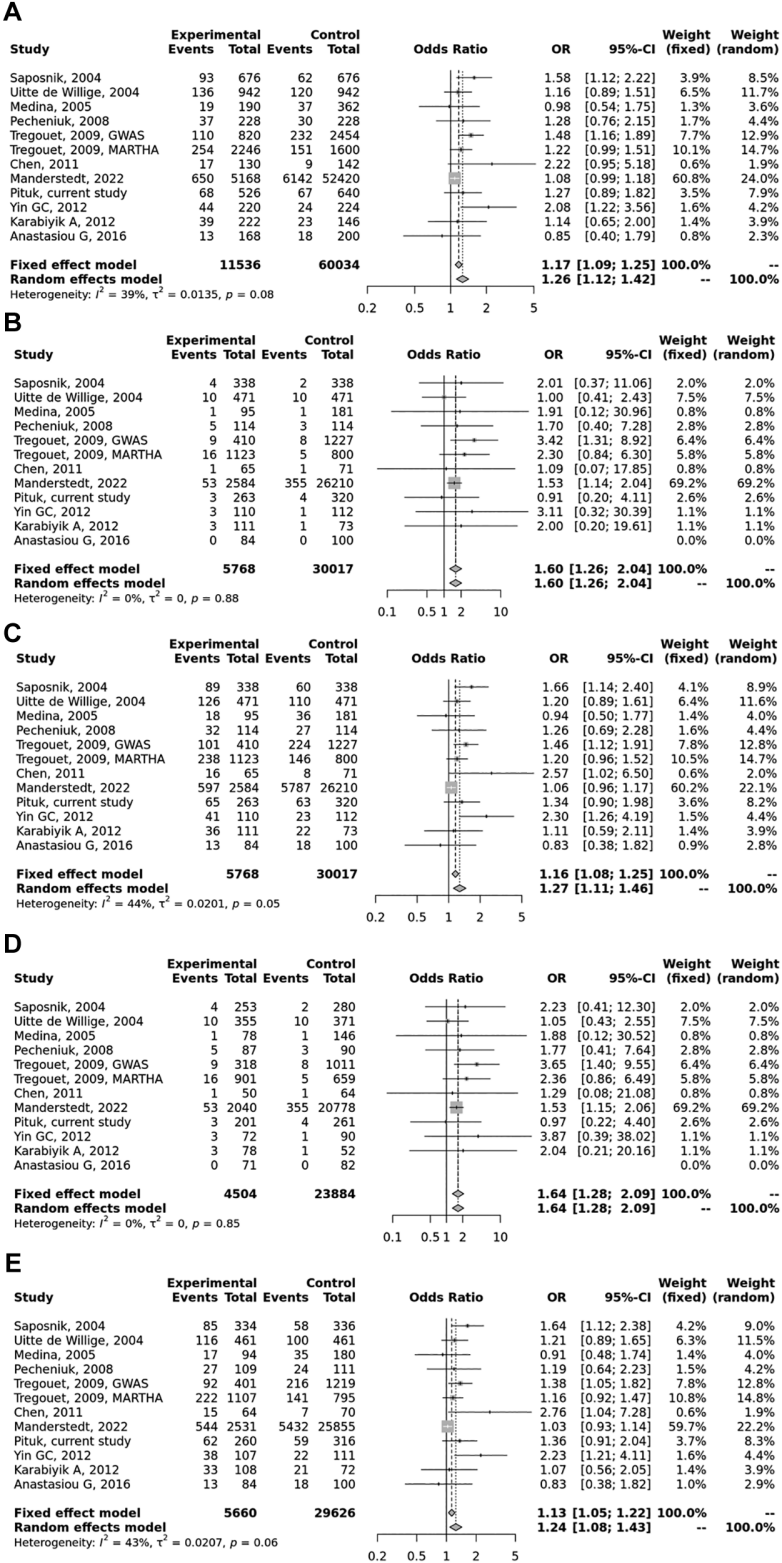
Pooled OR and 95% CI for the included studies for the association between the *PROCR* rs867186 genotypes and venous thromboembolism (VTE). (**A**) Allele contrast model (G vs. A). (**B**) Recessive model (GG vs. GA + AA). (**C**) Dominant model (GG + GA vs. AA). (**D**) GG vs. AA model. (**E**) GA vs. AA model. The square size represents the effect size of individual studies. The location of the squares reflects to the OR of the corresponding individual study and the length of their vertical lines describe the appropriate confidence interval. The diamond represents the overall or summary effect.

**Figure 3:**
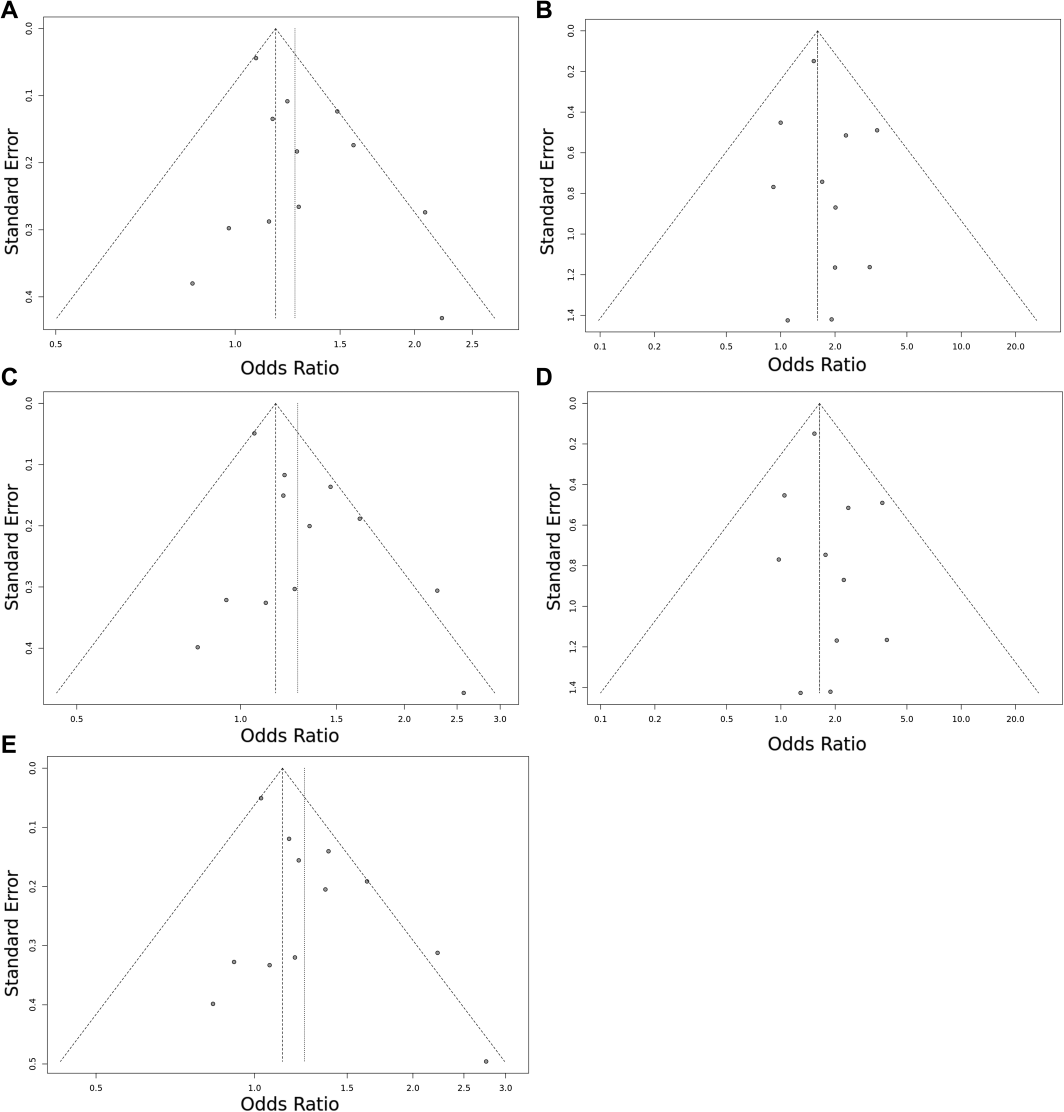
Funnel plot of the EPCR p.Ser219Gly polymorphism and VTE risk in the overall study population. (**A**) Allele contrast model (G vs. A). (**B**) Recessive model (GG vs. GA + AA). (**C**) Dominant model (GG + GA vs. AA). (**D**) GG vs. AA model. (**E**) GA vs. AA model. The points represent the individual studies. Horizontal axis represents the OR and vertical axis represents the standard error of the individual studies. The points, which are in the upper part of the triangle represent the large studies with small standard error.

**Figure 4:**
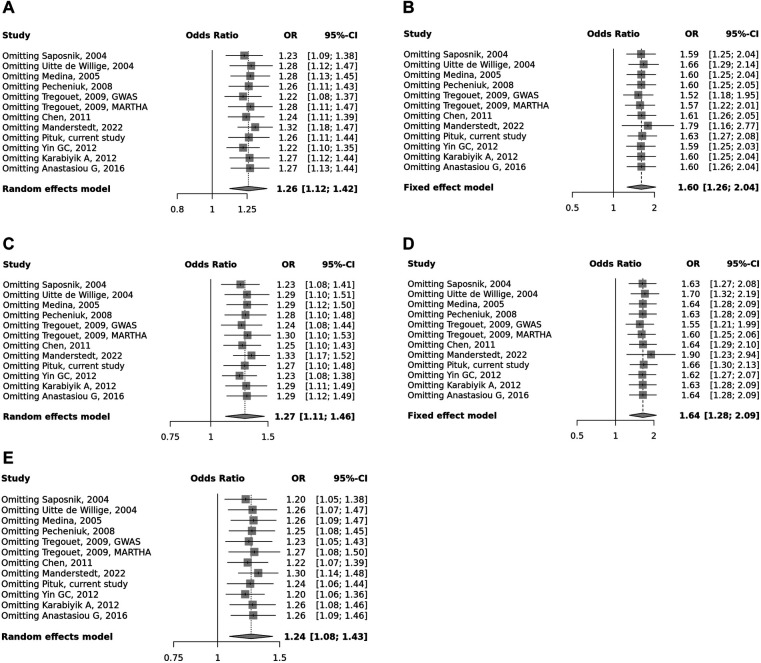
Summarized sensitivity analysis of association between *PROCR* rs867186 polymorphism and VTE. (**A**) Allele contrast model (G vs. A). (**B**) Recessive model (GG vs. GA + AA). (**C**) Dominant model (GG + GA vs. AA). (**D**) GG vs. AA model. (**E**) GA vs. AA model. The square size represents the effect size of individual studies. The location of the squares reflects to the OR of the corresponding individual study and the length of their vertical lines describe the appropriate confidence interval.

Overall, we found 53 potentially relevant candidate publications by searching the given literature database. The study selection process has been depicted in PRISMA diagram (Figure 1). For the current meta-analysis, we finally selected 11 studies (18–27). In the Identification phase, *n* = 19 studies were excluded due to duplication. In the screening phase, *n* = 20 studies were excluded. The excluded studies were: two *in vitro* studies involving cell lines, one study in an animal model, three meta-analyses, one study from which primary data could not be extracted, four studies with other gene mutations, and nine studies with different outcomes (acute myeloid leukemia, cerebral thrombosis, CHD patients, multiple myeloma, ovarian cancer patients, and unprovoked VTE). In the eligibility phase, four additional studies were excluded. Among them, two did not meet HWE (41, 42), and one other study had a similar study population and design as previously published by authors (43). One study was excluded because publication bias was detected (44). After the quality assessment and data extraction process, the total number of cases and controls were 5,768 and 30,017, respectively. (Table 3) After pooling all the selected studies, our current meta-analysis found a statistically significant association between EPCR p.Ser219Gly polymorphism and VTE under all applied genetic models (dominant model: OR = 1.27, 95% CI: 1.11–1.46; recessive model: OR = 1.60, 95% CI: 1.26–2.04; GG vs. AA contrast model: OR = 1.64, 95% CI: 1.28–2.09; GA vs. AA contrast model: OR = 1.24, 95% CI: 1.08–1.43) (Table 4) (Figures 2A–E). The between-study heterogeneity was low under all examined genetic models in our study. However, this may be caused by the small number of GG subjects, which can introduce a bias into this calculation and the true between-study heterogeneity is hidden, as previously published (13, 45). There were no significant differences among the included studies in the Egger test under the allele contrast (G vs. A) (*p* = 0.07), the recessive model (GG vs. GA + AA) (*p* = 0.58), the dominant model (GG + GA vs. AA) (*p* = 0.05), and the GG vs. AA contrast model (*p* = 0.43). Even the funnel plot—the plot of the log-odds ratio against the reciprocal of its standard error—did not demonstrate publication bias in these genetic models. The Egger regression asymmetry test has shown a significant difference under the GA vs. AA contrast model (*p* = 0.04), and the funnel plot suggested a publication bias (Figure 3). The sensitivity analysis showed no significant change in the pooled OR upon omitting individual studies (Figure 4).

**Table 3:** Characteristics of the eligible studies of the association between the *PROCR* rs867186 genotypes and VTE.

		VTE			Control			Deviation from Hardy–Weinberg equilibrium
Included study^a^	Region	AA	AG	GG	AA	AG	GG	*χ*^2^ *p*-value
Saposnik et al. (2004) (18)	France	249	85	4	278	58	2	0.58
Uitte de Willige et al. (2004) (19)	Netherland	345	116	10	361	100	10	0.33
Medina et al. (2005) (20)	Spain	77	17	1	145	35	1	0.47
Pecheniuk et al. (2008) (21)	USA	82	27	5	87	24	3	0.40
Tregouet et al. (2009), GWAS (22)	France	309	92	9	1,003	216	8	0.32
Tregouet et al. (2009), MARTHA (22)	France	885	222	16	654	141	5	0.38
Chen et al. (2011) (23)	China	49	15	1	63	7	1	0.15
Manderstedt et al. (2022) (24)	Sweden	1987	544	53	20,423	5,432	355	0.77
Pituk et al., current case–control study	Hungary	198	62	3	257	59	4	0.77
Yin et al. (2012) (25)	China	69	38	3	89	22	1	0.78
Karabiyik et al. (2012) (26)	Turkey	75	33	3	51	21	1	0.47
Anastasiou et al. (2016) (27)	Greece	71	13	0	82	18	0	0.32

^a^ The included studies were identified by the author's name, year of publication, study name, and reference number.

**Table 4:** Summary table of the meta-analysis of association between the *PROCR* rs867186 genotypes and VTE.

Genetic model	Number of studies	Test of association			Heterogeneity			Egge*r*'s test
		OR	95% CI	*p*-value	Model	*p*-value	*I* ^2^	*p*-value
Allele contrast (G vs. A)	12	1.26	1.12–1.42	0.0001	Random	0.084	0.39	0.07
Recessive model (GG vs. GA + AA)	11^a^	1.60	1.26–2.04	0.0001	Fixed	0.881	0.00	0.58
Dominant model (GG + GA vs. AA)	12	1.27	1.11–1.46	0.0006	Random	0.052	0.44	0.05
GG vs. AA	11^a^	1.64	1.28–2.09	0.0001	Fixed	0.851	0.00	0.43
GA vs. AA	12	1.24	1.08–1.43	0.0024	Random	0.056	0.43	0.04

^a^ In one of the selected studies, the GG genotype was absent.

### 3.3. Reproducibility study

By definition, reproducibility is the precision of the measurement by different research groups and different experimental settings (46). In our reproducibility study, we wanted to analyze whether the results of the meta-analysis remain the same with a different statistical software. We compared the data obtained by our method (a new, online MetaGenyo Statistical Analysis System software) to those obtained and published by Dennis et al. using STATA software version 11 (13). In the comparison study, we included only those studies that were included in the analysis of Dennis et al. We compared the deviation from HWE, the results of the test of association (OR and 95% CI), and between-study heterogeneity (*I*^2^ and Cochran *Q p*-value). We found no discrepancies in the meta-analysis study results obtained by STATA or MetaGenyo (Tables 5, 6), and the detected deviation in percentage was 0%.

**Table 5:** Details of the reproducibility study between STATA version 11 software and a new online MetaGenyo Statistical Analysis System software.

		VTE			Control			Deviation from Hardy–Weinberg equilibrium—MetaGenyo	Deviation from Hardy–Weinberg equilibrium—STATA
Included study	Region	AA	AG	GG	AA	AG	GG	*χ*^2^ *p*-value	*χ*^2^ *p*-value
Medina (2004)	Valencia, Spain	291	62	2	327	74	0	0.0418	0.04
Saposnik (2004)	France	249	85	4	278	58	2	0.5819	0.58
Uitte de Willige (2004)	Netherland	345	116	10	361	100	10	0.3286	0.33
Medina (2005)	Spain	77	17	1	145	35	1	0.4705	0.47
Navarro (2008)	Valencia, Spain	58	24	2	128	21	0	0.3548	0.35
Pecheniuk (2008)	USA	82	27	5	87	24	3	0.4002	0.40
Tregouet (2009), GWAS	France	309	92	9	1,003	216	8	0.3225	0.32
Tregouet (2009), MARTHA	France	885	222	16	654	141	5	0.3793	0.38
Yamagishi (2009)	LITE study	417	72	7	844	158	14	0.0384	0.04
Chen (2011)	China	49	15	1	63	7	1	0.153	0.15
Heit (2011)	China	978	264	28	1,029	257	16	0.9917	0.99

**Table 6:** Comparison table of the previously published meta-analysis of association between the *PROCR* rs867186 genotypes and VTE and simulated results using MetaGenyo Statistical Analysis System software.

Genetic model	Test of association				Heterogeneity			
	MetaGenyo	STATA	MetaGenyo	STATA	MetaGenyo	STATA	MetaGenyo	STATA
	OR	OR	95% CI	95% CI	*I* ^2^	*I* ^2^	Cochran *Q p*-value	Cochran *Q p*-value
GA vs. AA	1.2087	1.21	1.0465–1.3959	1.05–1.40	0.4309	43	0.0627	0.063
GG vs. AA	1.8138	1.81	1.2847–2.5608	1.29–2.56	0	0	0.6945	0.694
Dominant model (GG + GA vs. AA)	1.2460	1.25	1.0767–1.4420	1.08–1.44	0.4719	47	0.0411	0.041
Recessive model (GG vs. GA + AA)	1.7546	1.76	1.2437–2.4756	1.24–2.48	0	0	0.7393	0.739

The selected model was a random-effect model.

## 4. Discussion

As thrombophilia itself is a complex condition, the risk of first thrombosis or recurrence after a thrombotic episode should be calculated individually based on several pieces of evidence. One should collect data on the status of the players in the coagulation and anticoagulation processes and of environmental and acquired risk factors; after putting the pieces in the puzzle together, a final conclusion on thrombotic risk could be drawn at the personal level. Identifying the risk of recurrent thrombosis is an unmet need for several thrombophilia to decipher which patient should continue anticoagulant therapy. In our study, an elevated, however, statistically not significant VTE recurrence risk was associated with rs867186, which is worthy of further investigation in larger studies.

It is difficult to measure so many coagulation factors’ levels and perform functional assays of many proteins involved in the coagulation/anticoagulation balance since these assays are time-consuming and expensive. Nowadays, by using next-generation sequencing (NGS) technology, genetic testing has become more and more reasonable and widely available. The whole genome or exome sequencing is still not a reality, even not necessary in thrombophilia if a well-defined panel of candidate genes is created. To save time and cost, and to avoid false-positive interpretations, the so-called “thrombophilia gene panel” should include only those genes, whose alterations have proven biological consequences. In the case of *PROCR* rs867186, the level of sEPCR is elevated, as it was published by several studies and also confirmed by *in vitro* studies. Qu et al. demonstrated that in the case of stable cell lines expressing the p.219Gly variant of EPCR, the shedding of EPCR from the cell membrane was five- to sevenfold higher as compared to the p.219Ser variant after phorbol-12-myristate 13-acetate (PMA) stimulation. This led to higher sEPCR levels in the conditioned media of p.219Gly cells. They also demonstrated that the A3 haplotype (which includes the p.219Gly variant) is not only associated with an increased sEPCR level but is also associated with decreased PC activation (47). Ireland et al. showed an increased basal release of p.219Gly sEPCR coupled with higher thrombin generation. This suggested that less membrane-bound EPCR would be available for efficient PC activation (48).

In clinical studies, elevated sEPCR levels were detected in individuals carrying the p.219Gly allele, and it was associated with increased risk of thrombosis. In the study by Anastasiou et al., the sEPCR levels were not only elevated in individuals with the p.219Gly variant but they were also significantly higher in carrier patients with venous thrombosis as compared to control carriers (27). On the other hand, they found that the EPCR gene p.Ser219Gly gene polymorphism was not associated with an increased risk of thrombosis in a Greek population, most probably the gene polymorphism associated with an earlier development phase of thrombosis. In eight studies included in the meta-analysis of Dennis et al., a significant increase in sEPCR levels were associated with the p.Ser219Gly gene polymorphism (18, 19, 23, 41–43, 48, 49). The rs867186 and also rs8119351 variants of *PROCR* have an effect on PC levels. It was demonstrated by Athanasiadis et al. that they were responsible for 10.27% and 9.56% of the variance in PC plasma levels, respectively (16).

The genotype frequencies of EPCR p.Ser219Gly polymorphism were 75% (AA), 24% (AG), and 1% (GG) in the cases, and 81% (AA), 18% (AG), and 1% (GG) in the controls in our case–control study; and carriership of the “G” allele was without a significant effect on the risk of VTE (OR 1.339; 95% CI: 0.904–1.984). The majority of the case–control studies conducted by others and included in our meta-analysis, however, described a significant risk-increasing effect of rs867186 in their whole population (i.e., not in subgroup analysis). In the small study by Chen et al. investigating *n* = 65 cases and *n* = 71 controls, a significant risk of rs867186 was calculated in the “GA” vs. “AA” model (OR 2.75; 95%CI: 1.04–7.30) in the Chinese population (23). Significant VTE risk-increasing effect of this polymorphism was also found in another Chinese study (OR 1.912; 95% CI: 1.064–2.818 in the dominant model) (25). In a French population (*n* = 338 cases and *n* = 338 controls), the rs867186 increased the risk of VTE even in the multivariate analysis including age, sex, FV Leiden, and FII20210A (OR 1.8; 95% CI: 1.2–2.6) (18). In an Egyptian population, the carriership of the “G” allele was associated with an increased risk of VTE fourfold in the dominant model by investigating *n* = 90 cases and *n* = 90 controls (44).

In a small Turkish population (*n* = 111 cases and *n* = 73 controls), the rs867186 was without a significant VTE risk-increasing effect, although the sEPCR level was elevated in “GG” homozygous individuals (26). Similarly, in the study by Pecheniuk et al., including white and non-white subjects (*n* = 114 cases and *n* = 114 controls), no effect of this polymorphism on the risk of VTE was described (21). Uitte de Willige et al. did not find a significant association of this polymorphism with the risk of VTE either in a Dutch population involving *n* = 474 cases and *n* = 474 sex- and age-matched controls (19). They have described, however, that the low level of sEPCR was protective against VTE in their population. In a nested case–control study involving non-white and white subjects, the rs867186 was without effect on the risk of VTE (OR 0.93; 95% CI: 0.70–1.25) (42). The polymorphism, however, increased the sEPCR level, which was not associated with VTE in their study. Subgroup analysis was not performed in these studies.

In a Spanish study by Medina et al., with *n* = 95 VTE cases and *n* = 181 controls, all carriers of FV Leiden were investigated, and the rs867186 did not influence the risk of VTE (20). Even the adjustment for sex, age, FII20210A, and the presence of the other EPCR polymorphism (rs9574) did not modify the ORs. On the contrary, although there was no effect of rs867186 on the risk of VTE in the Greek population, the presence of the “G” allele was more prevalent among patients who developed VTE at younger age below 35 years (27). In a recent study, however, the rs867186 increased the risk of VTE in a large Swedish population, including middle-aged and older adults, where the HR was 1.5 (95% CI: 1.1–1.9) in “GG” homozygous individuals (*n* = 2,584 VTE cases and *n* = 26,201 without developing VTE) (24). Saposnik et al. demonstrated that the VTE risk-increasing effect of rs867186 was attributed to its effect in the male subgroup (18). In our study, rs867186 increased the risk of VTE in individuals who were wild type for rs8119351 (OR 3.2; 95% CI: 1.372–7.465), which remained significant in the multivariate model.

Based on these studies, the question is still open, whether to quantify sEPCR levels or simply determine the genotype of *PROCR* rs867186 from the point of view of first VTE and VTE recurrence. The level of sEPCR might be modified not only by *PROCR* genotype but also by different genetic and environmental factors. The reason for heterogeneity in different studies investigating the risk conferred by rs867186 and sEPCR levels might be the difference among populations in terms of influencing factors. It would be, therefore, beneficial to determine the factors—genetic and environmental—which have an effect on sEPCR levels. However, it should be taken into consideration that changes in sEPCR levels may also be consequences of different medical conditions, like thrombotic diseases, rather than being a risk factor. Large prospective studies could answer for the question, whether *PROCR* genotype or sEPCR levels are better predictors for VTE risk.

Due to heterogeneity in the results of the individual studies, we performed a meta-analysis in order to clarify the effect of rs867186 on the risk of VTE. Our meta-analysis of *n* = 5,768 VTE cases and *n* = 30,017 controls found a significant association between the EPCR gene p.Ser219Gly and VTE, under the dominant model (OR = 1.27, 95% CI: 1.11–1.46, *p* = 0.0006), the recessive model (OR = 1.60, 95% CI: 1.26–2.04, *p* = 0.0001), the GG vs. AA contrast model (OR = 1.64, 95% CI: 1.28–2.09, *p* = 0.0001), and the GA vs. AA contrast model (OR = 1.24, 95% CI: 1.08–1.43, *p* = 0.002). In the previous meta-analysis conducted by Dennis et al., including a smaller number of cases and controls (*n* = 4,821 cases and *n* = 6,070 controls), the main message was similar to our present analysis (OR 1.22; 95% CI: 1.11–1.33) (13). In the second meta-analysis by Li et al., *n* = 4,440 cases and *n* = 5,054 controls were analyzed, and they also found a significantly elevated risk of VTE in carriers of the “G” allele of the rs867186 (OR 1.63; 95% CI: 1.30–2.04). Here, we confirmed the results of these two previous meta-analyses. Moreover, we validated the novel, online free-access MetaGenyo Statistical Analysis System software by a reproducibility study. Based on our findings, this online platform is acceptable for meta-analysis in case of genetic epidemiology studies.

The risk conferred by the rs867186 is significantly increased but mild in all meta-analyses, including ours. However, in the concept of personalized medicine including personalized risk assessment, every factor might have an impact. Collection as many pieces of information including environmental and genetics as it is possible may serve as a basis of the personalized risk management of VTE. The availability of high-throughput genetic methods, like next-generation sequencing technology, supports the acquisition of large-scale genetic data from the patients. Upon performing gene panel sequencing, the simultaneous investigation of candidate genes can be executed. The *PROCR* is currently not included in the panel of the Thrombogenomics platform (50), even missing from the gene panel list of the ISTH SSC on Genomics in Thrombosis and Hemostasis Tier 1 genes (https://www.isth.org/page/GinTh_GeneLists). Including *PROCR* in the gene panels may help collect more experience of the role of its variants in VTE not only from the point of view of absolute VTE risk but also from the point of view of VTE recurrence and risk modifying effect in individuals with PC deficiency.

There were only two cases detected with PC deficiency in our study group during the recruitment period. The first patient had the c.811C > T (p.Arg271Trp) and the second carried the c.169C > T (p.Arg57Trp) mutation both in heterozygous forms. These mutations are listed in various databases as pathogenic/likely pathogenic variants and associated with PC deficiency and thrombotic phenotype. Both individuals carried the rs867186 in heterozygous form. The first patient had one thrombotic episode at the age of 29 years; no recurrence has been registered until now. The second had venous thrombosis first at the age of 29 years and one recurrent event at the age of 33 years while on oral anticoagulant therapy. Upon including these two PC-deficient patients in the statistical analysis, no modifications in the results were observed. To study the modifying effect of *PROCR* polymorphisms on the PC levels and the risk of VTE, a study directly recruiting PC-deficient individuals would be beneficial. In a previous study, Fidalgo et al. investigated Portuguese PC-deficient families and suggested that rs867186 explained differences in PC plasma levels in individuals with the same *PROC* genotype (51), and very high PC levels were detected in a study by Pintao et al. in case of individuals homozygous for the *PROCR* rs867186 (52). Homozygosity for the rs867186 was hypothesized to be the underlying condition of extremely high PC levels in a large French cohort (53). These studies, however, did not describe the potential VTE risk modifying effect of EPCR polymorphisms in PC deficiency.

The strength of the current meta-analysis is that it updated the previous results with a large-scale study (24) and added the Hungarian case–control study results. In addition to that, we also performed a reproducibility study to ensure the validity of our results.

Our study had limitations that should be acknowledged. Our case–control study might have limited generalizability through the use of strict exclusion criteria regarding the selection of the control group. Therefore, we are not sure to what extent the absence of chronic diseases and arterial thrombosis might influence the results on the risk of EPCR polymorphism on VTE.

Our meta-analysis detected potential publication bias under the GA vs. AA contrast model. Furthermore, we must consider the potential selection bias and possible misclassification of the genotype and phenotype results regarding the individual studies. We were not able to perform any further subgroup analysis because of the small group number of similar ethnicities. On the other hand, due to the non-standardized data availability of the individual studies included in our meta-analysis, we could also not create a dedicated analysis by other factors such as age, gender, smoking, alcohol consumption, and other environmental or lifestyle factors.

## Data Availability

The raw data supporting the conclusions of this article will be made available by the authors, without undue reservation.
